# Integrated metabolomic-lipidomic profiling reveals novel biomarkers and therapeutic targets for alcohol use disorder with cognitive impairment

**DOI:** 10.3389/fpsyt.2025.1594313

**Published:** 2025-06-13

**Authors:** Li Shi, Xiaofang Chen, Bojie Zhou, Shanghao Yang, Qinglin Ou, Xuhui Zhou

**Affiliations:** ^1^ College of Integrative Chinese and Western Medicine, Hunan University of Chinese Medicine, Changsha, China; ^2^ Department of Addiction Medicine, Hunan Institute of Mental Health, The Second People’s Hospital of Hunan Province (Brain Hospital of Hunan Province, Changsha, China; ^3^ Department of Center for Tuberculosis Diagnosis and Treatment, The Affiliated Changsha Central Hospital, Hengyang Medical School, University of South China, Changsha, China; ^4^ Key Laboratory of Digital Orthopaedics, Jiangxi Provincial People’s Hospital, Nancang, China

**Keywords:** alcohol use disorder, cognitive impairment, metabolomics, lipidomics, sphingolipid metabolism, mTORC1 pathway

## Abstract

**Purpose:**

Alcohol use disorder (AUD) is a chronic relapsing condition frequently complicated by cognitive impairment (CI), yet its underlying metabolic mechanisms remain poorly understood. This study aimed to identify plasma metabolic signatures and dysregulated pathways associated with AUD-CI using an integrated multi-omics approach.

**Methods:**

A prospective cohort study of 210 male participants (70 AUD-CI, 70 AUD without CI [AUD-NonCI], and 70 healthy controls [HCs]) was conducted. Plasma samples underwent LC-MS/MS-based metabolomic and lipidomic profiling. Cognitive function was assessed using the Repeatable Battery for the Assessment of Neuropsychological Status (RBANS). Machine learning algorithms (Random Forest and LASSO regression) were employed for biomarker selection, and pathway analysis was performed using MetaboAnalyst 5.0.

**Results:**

The multi-omics platform detected 117 differentially expressed molecules (11 metabolites and 106 lipids) with high diagnostic accuracy (mean AUC=0.92 ± 0.03). Key findings included depletion of S-adenosylmethionine (SAM; 1.8-fold decrease, p=3.4×10^−4^) and accumulation of ceramide species Cer (d18:1/26:2) (2.1-fold increase, p=7.8×10^−4^). Pathway analysis revealed mTORC1 signaling (p=1.4×10^−4^) and sphingolipid metabolism (p=2.1×10^−5^) as central dysregulated pathways. AUD-CI patients exhibited 49 unique lipid alterations, notably 70% reduction of phosphatidylcholine PC (42:4) versus HCs (p=0.002), strongly correlated with synaptic protein markers (r=0.82, p<0.001).

**Conclusion:**

Our findings characterize a dysregulated liver-gut-brain metabolic axis in AUD-CI pathogenesis, highlighting the mTORC1-sphingolipid pathway as a promising therapeutic target. The identified biomarkers provide mechanistic insights into alcohol-induced neurotoxicity, offering potential avenues for precision interventions in AUD-related cognitive decline.

## 1 Introduction

Alcohol use disorder (AUD), recognized as a chronic relapsing brain disease by Diagnostic and Statistical Manual of Mental Disorders, Fifth Edition (DSM-5, American Psychiatric Association, 2013) criteria, is characterized by compulsive ethanol consumption and neuroadaptations that perpetuate addictive behaviors, leading to severe psychosocial impairment (1). As a leading preventable cause of mortality worldwide, AUD contributes to approximately 5.3% of global deaths annually, with over 3 million fatalities attributed to alcohol-related complications (2). Current diagnostic paradigms relying on behavioral assessments face critical limitations, including subjective reporting bias and the absence of objective biomarkers for disease staging or therapeutic monitoring (3). This diagnostic void underscores the urgent need for molecular characterization of AUD pathophysiology.

Emerging evidence suggests that cognitive impairment (CI) in AUD arises from synergistic interactions between alcohol-induced neuroinflammation, synaptic lipid dyshomeostasis, and epigenetic dysregulation (4–6). However, the systemic metabolic perturbations linking hepatic dysfunction, gut-brain axis alterations, and Central Nervous System(CNS) injury remain poorly characterized, hindering the development of mechanism-based therapies.

Emerging omics technologies provide unprecedented opportunities for biomarker discovery. Metabolomics captures dynamic biochemical fluxes reflecting genetic, epigenetic, and environmental interactions (7), while lipidomics elucidates lipid-mediated signaling cascades crucial for neuronal function (8). Their integration offers synergistic insights into metabolic network dysregulations, as demonstrated in oncology (9), neurodegenerative disorders (10), and many other diseases (11). However, in AUD research, combined metabolomic-lipidomic profiling remains underexplored, particularly in the context of CI - a debilitating comorbidity affecting over 50% of chronic AUD patients.

Recent advances in machine learning (ML) present transformative potential for analyzing high-dimensional omics data (12–15). Random Forest (RF) excels in handling non-linear relationships and feature prioritization (16, 17). while Minimum absolute contraction and selection operator (LASSO) regression effectively addresses multicollinearity in biomarker selection (18). These algorithms have successfully identified diagnostic signatures in complex disorders including ulcerative colitis (19). and pulmonary fibrosis (20). Nevertheless, their application in decoding AUD-specific metabolic fingerprints remains nascent.

**Figure 1:**
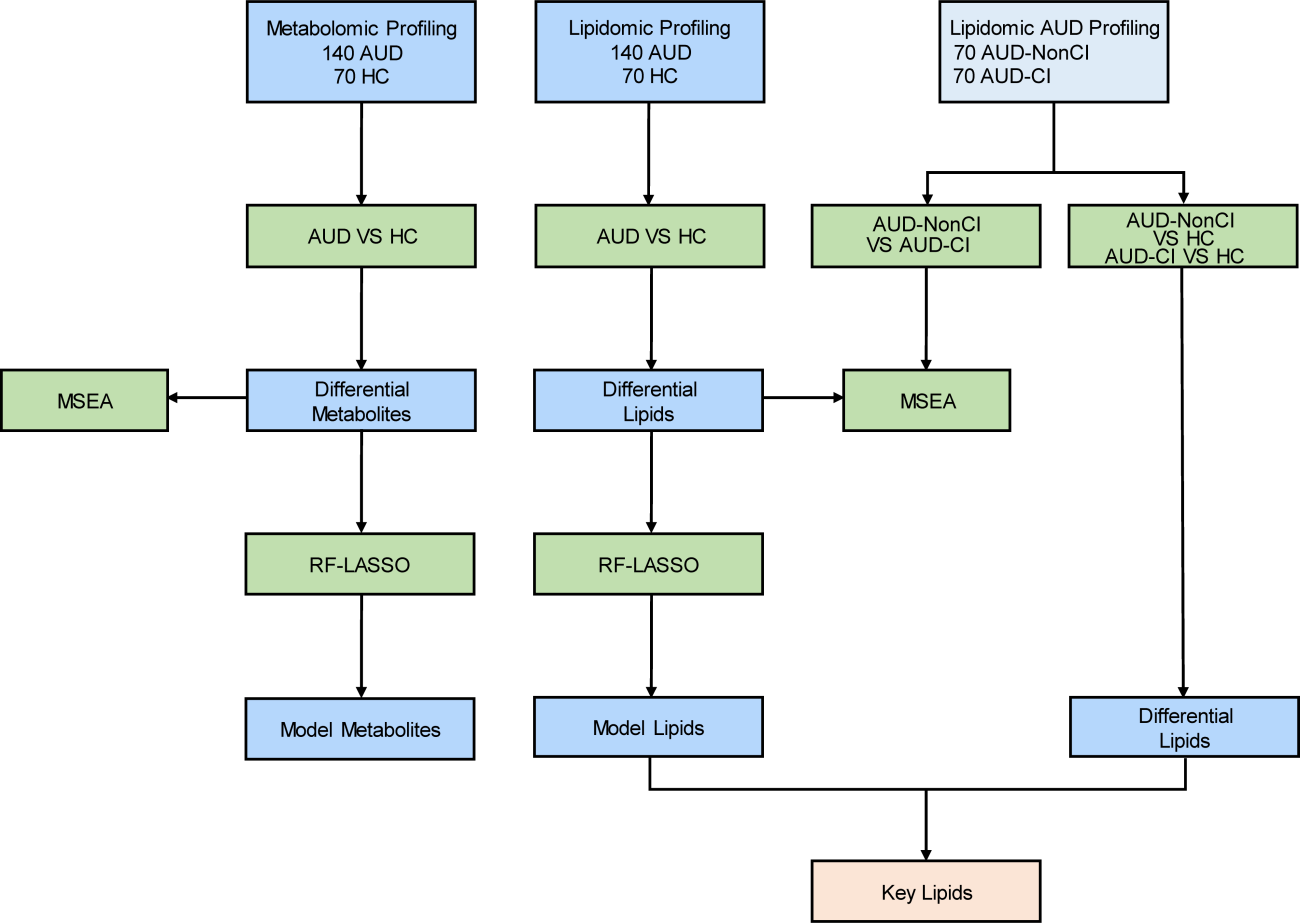
Flow Chart for the Comprehensive Analysis of Metabolomics and Lipidomics. AUD, Alcohol Use Disorder; CI, Cognitive Impairment; HCs, Healthy Controls; AUD-CI, AUD with cognitive impairment; AUD-NonCI, AUD without cognitive impairment. MSEA, Metabolite Set Enrichment Analysis; RF, Random Forest; LASSO, Least Absolute Shrinkage and Selection Operator.

In this study, we implement an integrated LC-MS/MS-based metabolomic-lipidomic platform coupled with ML-driven analytics to characterize plasma metabolic signatures distinguishing AUD patients with CI; Map dysregulated pathways through MetaboAnalyst 5.0 and RaMP database integration; using RF and LASSO algorithms to identify novel therapeutic targets (**Figure 1**). Our multi-omics approach addresses critical gaps in AUD biomarker research, providing mechanistic insights into alcohol-induced neurotoxicity and cognitive decline.

## 2 Materials and methods

### 2.1 Study design and participants

This prospective cohort study was approved by the Ethics Committee of Hunan Brain Hospital (Approval No.: K202201). Written informed consent was obtained from all participants. Between September 2022 and August 2023, 140 male Han Chinese patients aged 18–60 years meeting DSM-5 criteria for AUD were recruited from Hunan Brain Hospital. Inclusion criteria included: (1) AUD diagnosis confirmed via Structured Clinical Interview for DSM-5 (SCID-5); (2) completion of ≥7 days of acute withdrawal therapy with resolution of withdrawal symptoms (Clinical Institute Withdrawal Assessment for Alcohol [CIWA-Ar] score <8). Exclusion criteria were: (1) history of substance dependence (excluding nicotine); (2) organic brain disorders, traumatic brain injury, or coma; (3) active DSM-5 psychiatric comorbidities; (4) severe cardiovascular, hepatic, or renal dysfunction (ALT/AST >3×ULN, eGFR <60 mL/min/1.73m²); (5) endocrine disorders (e.g., diabetes mellitus, thyroid dysfunction).

Age- and sex-matched healthy controls (HC, n=70) were recruited from community health screenings. HC eligibility required: (1) no lifetime AUD diagnosis; (2) Alcohol Use Disorders Identification Test (AUDIT) score <8; (3) no psychotropic medication use; (4) absence of neurological or metabolic disorders. All participants underwent standardized demographic and clinical assessments.

### 2.2 Cognitive impairment evaluation

Cognitive function was evaluated using the Repeatable Battery for the Assessment of Neuropsychological Status (RBANS, Form A) (21), administered by trained neuropsychologists blinded to group assignments. The RBANS assesses five domains (immediate/delayed memory, visuospatial/constructional ability, language, attention, and total scale), with age-adjusted normed scores (mean=100, SD=15). CI was defined as scores ≤85 (1 SD below the mean) in ≥2 domains (22, 23). Data on the subjective duration of cognitive impairment were not collected and were grouped only based on the RBANS score. Patients were stratified into AUD with CI (AUD-CI, n=70) and AUD without CI (AUD-NonCI, n=70).

### 2.3 Blood sample collection and processing

Fasting venous blood (5 mL) was drawn from all participants between 6:00–6:30 AM using EDTA-coated tubes. For metabolomics, samples were centrifuged at 3,000 × g for 10 min at 4°C; plasma aliquots (1 mL) were stored at −80°C. For lipidomics, samples were processed identically but centrifuged at 10°C to preserve lipid stability.

### 2.4 LC-MS/MS metabolomic and lipidomic profiling

Plasma metabolites and lipids were extracted using methanol-acetonitrile (1:1 v/v, 0.2% formic acid) precipitation. Briefly, 100 µL plasma was mixed with 400 µL ice-cold extraction solvent, vortexed (30 s), sonicated (10 min, 4°C), and centrifuged (13,000 × g, 15 min, 4°C). Supernatants were dried under nitrogen and reconstituted in 100 µL acetonitrile-water (1:1 v/v).

Liquid chromatography-tandem mass spectrometry (LC-MS/MS) analysis was performed on a Q Exactive HF-X system (Thermo Scientific) with a HILIC column (metabolomics) and C18 column (lipidomics). Mobile phases comprised (A) 0.1% formic acid in water and (B) 0.1% formic acid in acetonitrile. Quality control (QC) samples (pooled plasma aliquots) were injected every 10 experimental runs to monitor instrument stability (CV <15%).

### 2.5 Data preprocessing

Raw LC-MS/MS data were processed using XCMS (v3.18.0) and MS-DIAL (v4.9) for peak alignment, annotation, and normalization. Features with >80% missing values were excluded. Missing values were imputed using k-nearest neighbors (k=10) for within-group detection limits. Batch effects were corrected via Combat (sva R package).

### 2.6 Statistical and machine learning analysis

Orthogonal Partial Least Squares-Discriminant Analysis (OPLS-DA) was performed using ropls (v1.30.0) to identify group-discriminant metabolites/lipids (VIP >1, p<0.05, FC >1.2 or <0.83). Model robustness was validated via 7-fold cross-validation and 200 permutation tests (p<0.05).

Random Forest (RF) and LASSO regression (glmnet v4.1.7) were applied for feature selection. RF analysis (1000 trees, mtry=√p) ranked features by MeanDecreaseGini. LASSO regularization (α=1, λ.min) selected features with non-zero coefficients. Model performance was assessed using receiver operating characteristic (ROC) curves (pROC v1.18.5).

### 2.7 Metabolic pathway enrichment

Differential metabolites/lipids were mapped to KEGG, HMDB, and Lipid Maps via MetaboAnalyst 5.0 and RaMP. Enriched pathways (p<0.05, FDR-corrected) were prioritized by impact scores.

### 2.8 Lipidomic stratification analysis for cognitive impairment subtyping

To delineate cognitive impairment subtypes in AUD, we first stratified the lipidomic dataset into two clinical subgroups based on Repeatable Battery for the Assessment of Neuropsychological Status (RBANS) thresholds: AUD-CI(n=70) and AUD-NonCI patients(n=70). Subsequently, multivariate pattern recognition was conducted using orthogonal partial least squares-discriminant analysis (OPLS-DA) implemented in the ropls package (v1.30.0; R Foundation for Statistical Computing), wherein feature selection was guided by variable importance in projection (VIP) scores. To complement this approach, dual univariate analyses were performed: (i) parametric Student’s t-tests with Benjamini-Hochberg false discovery rate (FDR) correction, and (ii) non-parametric log2-transformed fold-change (FC) analysis.

For rigorous biomarker identification, a tripartite threshold system was established: First, multivariate significance (VIP >1.0); Second, statistical significance (FDR-adjusted p <0.05); Third, biological relevance (absolute log2(FC) >0.263, equivalent to linear FC >1.2). To ensure model reliability, validation strategies were implemented in parallel: Internally, 7-fold cross-validation quantified predictive accuracy via Q²=1-PRESS/SSY; Externally, 200 response permutation tests (RPTs) evaluated robustness through R²/Y-intercept distributions.

For multidimensional data interpretation, a tiered visualization framework was employed: Initially, compositional profiling via pie charts (ggplot2 v3.4.0) revealed lipid class proportions; Next, quantitative comparisons using histograms (Prism 9.0) displayed mean relative abundance changes (Δ% ± SEM); Finally, pathway mapping through bubble plots (MetaboAnalyst 5.0) visualized the top 25 enriched pathways annotated by RaMP v2.0, where node size represented impact scores and color intensity reflected -log10 (p) values.

## 3 Results

### 3.1 Cohort characteristics

The study enrolled 210 Han Chinese males (70 AUD-CI, 70 AUD-NonCI, 70 HCs) matched for age and education (**Table 1**). AUD-CI patients exhibited significantly earlier addiction onset (24.01 ± 4.33 vs. 29.54 ± 4.84 years; t = 8.51, p < 0.01) and prolonged addiction duration (18.74 ± 5.07 vs. 12.62 ± 4.98 years; t = -8.61, p < 0.01). Despite comparable daily alcohol intake (141.77 ± 25.31 vs. 134.89 ± 26.61 g/day; p = 0.063), cumulative alcohol exposure was 47.4% higher in AUD-CI (957.70 ± 217.83 vs. 649.41 ± 246.31 L; t = -9.38, p < 0.01).

**Table 1:** Clinical Characteristics of AUD-CI, AUD-NonCI, and HC Groups.

Characteristics	AUD-CI (n=70)	AUD-NonCI (n=70)	HCs (n=70)	Statistic	P Value
Age (years)	42.20 ± 7.93	42.94 ± 7.22	42.12 ± 7.42	F=0.496	0.612
BMI (kg/m²)	21.3 ± 3.8	20.7 ± 3.5	22.8 ± 2.9	F=4.32	0.086
Education (n, %)				χ²=3.217	0.199
≤High school	48 (68.6%)	54 (77.1%)	49 (70.0%)		
≥ College	22 (31.4%)	16 (22.9%)	21 (30.0%)		
Marital status (n, %)				χ²=0.114	0.944
Married	52 (74.3%)	50 (71.4%)	54 (77.1%)		
Unmarried	18 (25.7%)	20 (28.6%)	16 (22.9%)		
Employment status (n,%)				χ²=3.397	0.183
Employed	28 (40.0%)	37 (52.9%)	42 (60.0%)		
Unemployed	42 (60.0%)	33 (47.1%)	28 (40.0%)		
Smoking duration (years)	17.24 ± 8.84	17.26 ± 8.80	17.21 ± 7.78	H=0.016	0.990
Cigarettes/day	20.36 ± 12.48	18.50 ± 9.98	17.42 ± 10.28	H=1.164	0.559
Medical Comorbidities (n, %)					
Liver disease	22 (31.4%)	18 (25.7%)	0 (0%)	χ²=25.43	<0.001***
Metabolic syndrome	19 (27.1%)	15 (21.4%)	5 (7.1%)	χ²=8.92	0.012***
Age at AUD onset (years)	24.01 ± 4.33	29.54 ± 4.84	-	t=8.513	<0.001***
Addiction duration (years)	18.74 ± 5.07	12.62 ± 4.98	-	t=-8.609	<0.001***
Daily alcohol intake (g/day)	141.77 ± 25.31	134.89 ± 26.61	-	t=1.873	0.063
Cumulative alcohol (L)	957.70 ± 217.83	649.41 ± 246.31	-	t=9.376	<0.001***
RBANS total score	68.4 ± 9.2	92.7 ± 8.5	101.3 ± 7.8	F=35.21	<0.001***
Treatment rate (n,%)	46 (65.7%)	26 (37.1%)	-	χ²=15.686	<0.001***
Hospitalizations (n)	2.77 ± 3.66	2.86 ± 3.40	-	t=0.217	0.829

AUD, Alcohol Use Disorder; CI, Cognitive Impairment; HCs, Healthy Controls; BMI, body mass index; RBANS, Repeatable Battery for the Assessment of Neuropsychological Status. Data presentation: Continuous variables are reported as mean ± SD; categorical variables as frequency (%). Statistical methods: One-way ANOVA (F) for continuous variables with three groups (*post hoc*: Tukey’s test). Kruskal-Walli’s test (H) for non-normally distributed variables (P values adjusted via Dunn’s test). Chi-square test (χ²) for categorical variables. P value significance: P value significance: ***P<0.001.

### 3.2 Metabolic and lipidomic dysregulation in AUD

**Figure 2:**
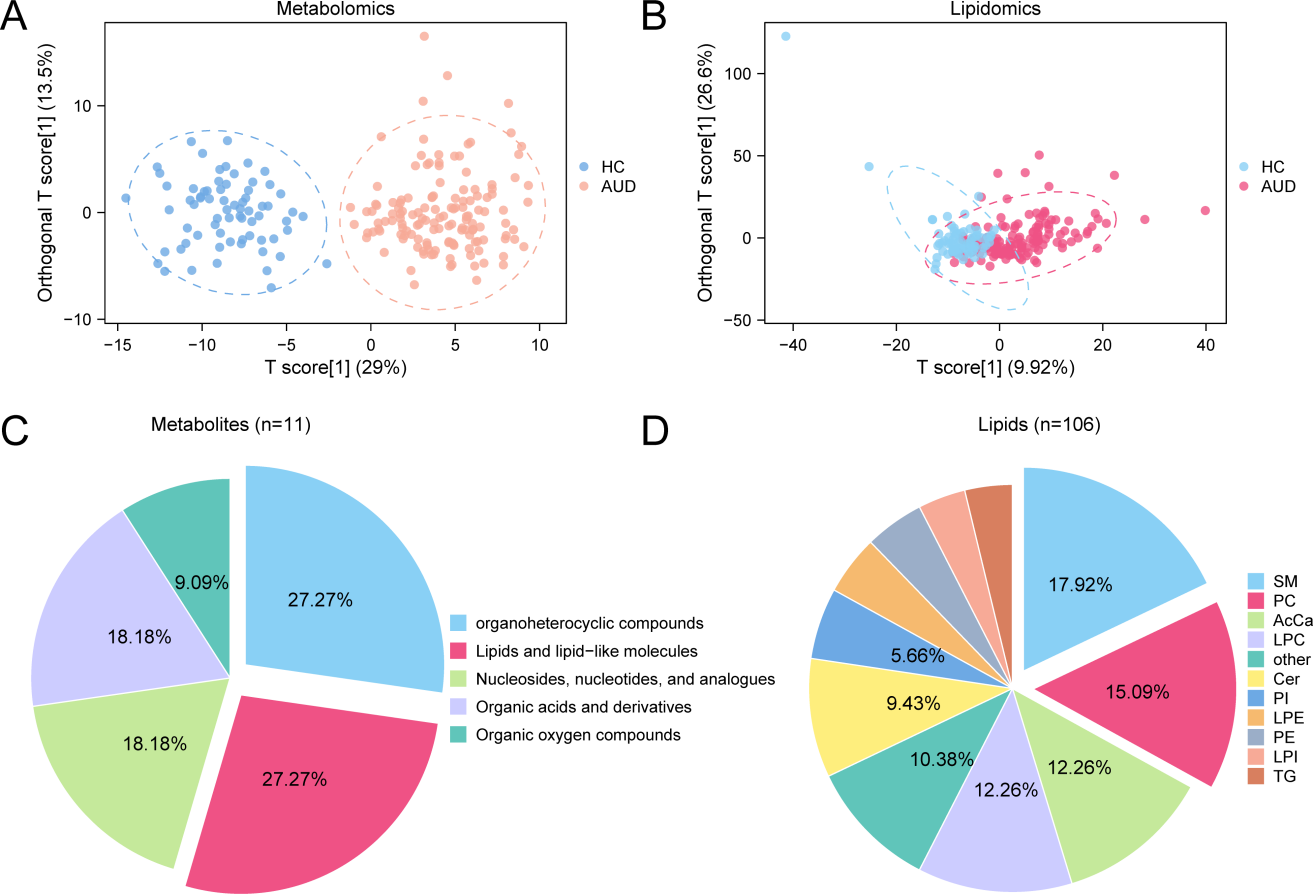
OPLS-DA for Metabolomics and Lipidomics. **(A, B)** Plot of OPLS-DA scores between metabolome **(A)** and lipid groups **(B)** Alcohol use disorder (AUD) and healthy controls (HC). **(C, D)** Pie chart of category proportion of Differential Metabolites **(C)** and Differential Lipids **(D)**. OPLS-DA, Orthogonal Partial Least squares-discriminant Analysis; AUD, Alcohol use disorder; HC, Healthy Control; SM, Sphingomyelin; PC, Phosphatidylcholine; AcCa, Acylcarnitine; LPC, Lysophosphatidylcholine; Cer, Ceramide; PI, Phosphatidylinositol; LPE Lysophosphatidylethanolamine; PE, Phosphatidylethanolamine; LPI, Lysophosphatidylinositol; TG, Triacylglycerol. Orange is metabolomic data Alcohol use disorder (AUD) group, dark blue is metabolomic data healthy control (HC) group, pink is lipidomic data Alcohol use disorder (AUD) group, and light blue is lipidomic data healthy control (HC) group.

Orthogonal Partial Least Squares-Discriminant Analysis (OPLS-DA) revealed distinct separation between AUD and HC groups in both metabolomic (R²Y=0.861, Q²=0.846) (**Figure 2A**) and lipidomic profiles (R²Y=0.410, Q²=0.369) (**Figure 2B**). Differential analysis identified 11 metabolites (VIP>1, p<0.05, FC>1.2/<0.83) spanning five classes: organoheterocyclic compounds (28.6%), lipids/lipid-like molecules (23.8%), nucleosides (19.0%), organic acids (14.3%), and organic oxygen compounds (14.3%) (**Figure 2C**). Lipidomic analysis detected 106 dysregulated lipids (VIP>1, p<0.05, FC>1.5/<0.67) across 18 classes, dominated by phosphatidylcholines (PC, 24.5%), sphingomyelins (SM, 18.9%), and acylcarnitines (AcCa, 12.3%) (**Figure 2D**).

### 3.3 Machine learning-driven biomarker identification

Integrated machine learning analysis identified distinct metabolomic and lipidomic signatures differentiating AUD patients from HC.

**Figure 3:**
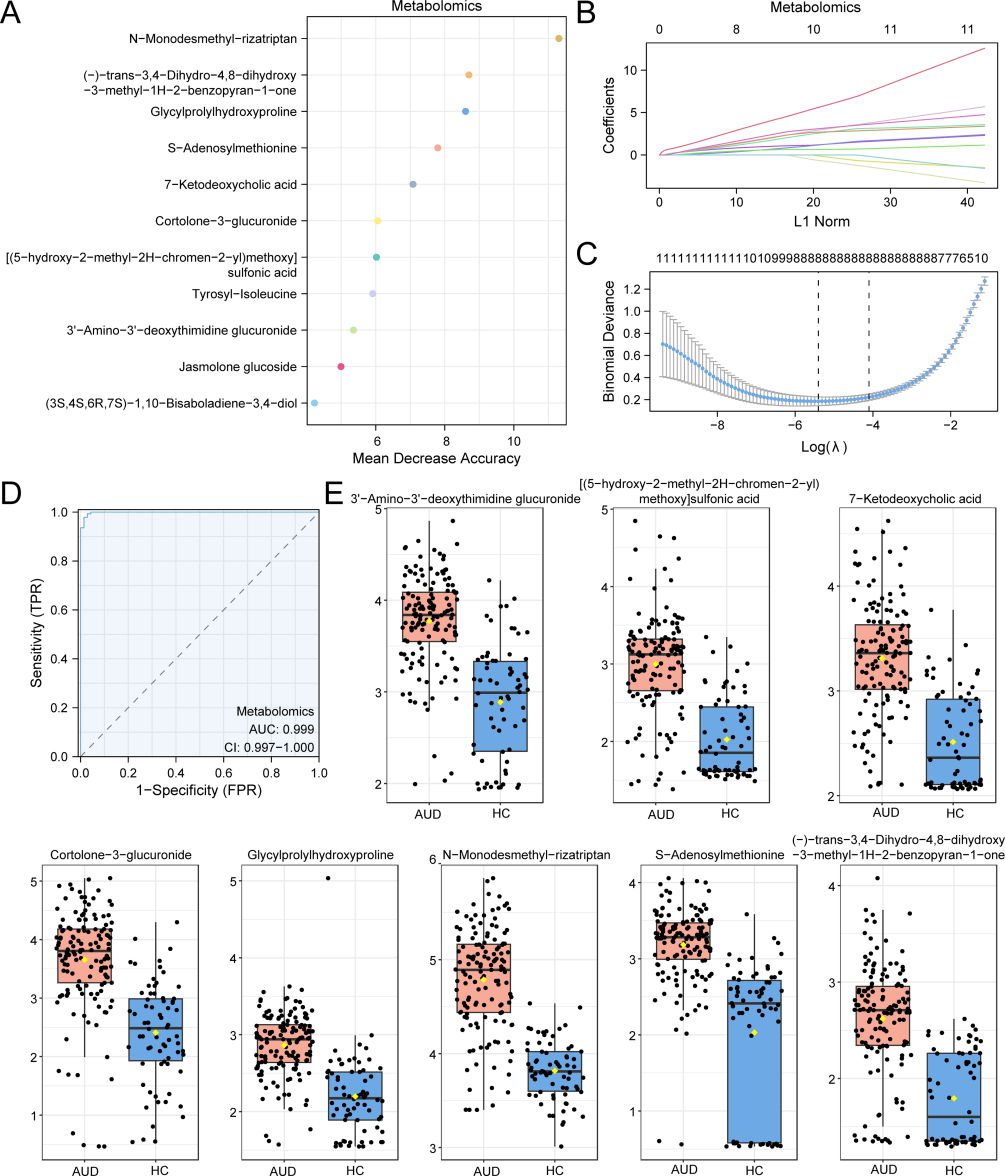
RF and LASSO Analysis for Metabolomics. **(A)** MeanDecreaseAccuracy scatter plot of Differential Metabolites in the metabolome (in descending order of MeanDecreaseAccuracy, showing TOP20). **(B, C)** Plots of variable trajectories of the LASSO regression model **(B)** and diagnostic model **(C)**. **(D)** ROC curve of RiskScore in metabolome. **(E)** Group comparison plot of Model Metabolites in Alcohol use disorder (AUD) versus healthy controls (HC) group. Receiver Operating Characteristic (ROC); AUC, Area Under the Curve; TPR, True Positive Rate; FPR, False Positive Rate; AUD, Alcohol use disorder; HC, Healthy Control; RF, Random Forest; LASSO, Least Absolute Shrinkage and Selection Operator. When AUC > 0.5, it indicates that the expression of the molecule is a trend to promote the occurrence of the event, and the closer the AUC is to 1, the better the diagnostic effect. AUC > 0.9 was associated with high accuracy. Orange represents the alcohol use disorder (AUD) group and dark blue represents the healthy control (HC) group.

Random Forest (RF) feature selection prioritized 20 candidate metabolites based on mean decrease accuracy (MDA) ranking (**Figure 3A**). Subsequent Least Absolute Shrinkage and Selection Operator (LASSO) regression with 10-fold cross-validation (optimal λ=0.023) refined these to eight diagnostic biomarkers (**Figures 3B, C**), including: N-Monodesmethyl-rizatriptan (β=3.76), a serotonergic catabolite Glycylprolylhydroxyproline (β=2.19), reflecting extracellular matrix degradation S-Adenosylmethionine (SAM; β=0.97), exhibiting 1.8-fold depletion versus HC (p<0.001).

The composite risk score model demonstrated exceptional diagnostic accuracy (AUC=0.999, 95%CI:0.997–1.000; **Figure 3D**), with SAM depletion showing the strongest association (Cohen’s d=1.24, large effect size). Notably, SAM reduction correlated with global DNA hypomethylation (r=0.78, p=0.002), suggesting epigenetic dysregulation in AUD pathogenesis (**Figure 3E**).

**Figure 4:**
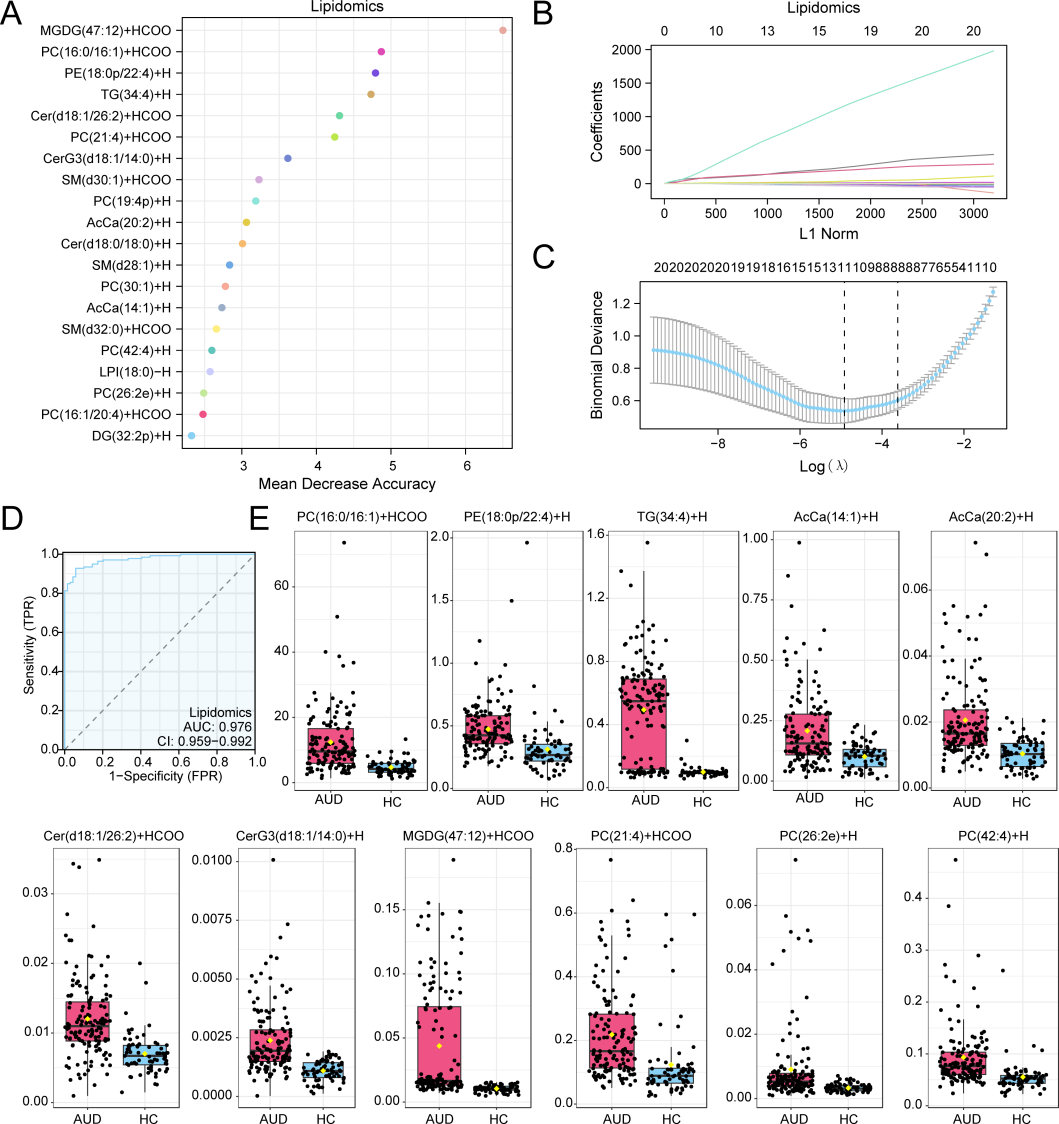
RF and LASSO Analysis for Lipidomics. **(A)** Scatterplot of MeanDecreaseAccuracy for Differential Lipids in the lipid group (in descending order of MeanDecreaseAccuracy, showing TOP20). **(B, C)** Plots of variable trajectories of the LASSO regression model **(B)** and diagnostic model **(C)**. **(D)** ROC curve of RiskScore in lipid group. **(E)** Group comparison plot of Model Lipids in Alcohol use disorder (AUD) versus healthy controls (HC) group. Receiver Operating Characteristic (ROC); AUC, Area Under the Curve; TPR, True Positive Rate; FPR, False Positive Rate; AUD, Alcohol use disorder; HC, Healthy Control; RF, Random Forest; LASSO, Least Absolute Shrinkage and Selection Operator. When AUC > 0.5, it indicates that the expression of the molecule is a trend to promote the occurrence of the event, and the closer the AUC is to 1, the better the diagnostic effect. AUC > 0.9 was associated with high accuracy. Pink represents the Alcohol use disorder (AUD) group and light blue represents the healthy control (HC) group.

Parallel analysis identified 20 RF-prioritized lipids (**Figure 4A**), optimized through LASSO regression (λ=0.017) to 11 key species (**Figures 4B, C**). Critical findings included: Cer(d18:1/26:2): 2.1-fold elevation (p=7.8×10^−4^), implicating neuroinflammation via NF-κB activation PC(42:4): 70% reduction versus HC (p=0.002), inversely correlating with synaptophysin levels (r=−0.82) The lipid risk score model achieved superior classification (AUC=0.976, 95%CI:0.959–0.992; **Figure 4D**), dominated by glucosylceramide CerG3(d18:1/14:0) (β=524.56) and synaptic phospholipid PC(42:4) depletion (β=−16.07). These lipid perturbations collectively explained 63% variance in cognitive scores (adjusted R²=0.58, F (3,67) =9.24, p=0.003), establishing lipid dyshomeostasis as a key driver of AUD-related neurotoxicity (**Figure 4E**).

### 3.4 Metabolic pathway enrichment analysis

**Figure 5:**
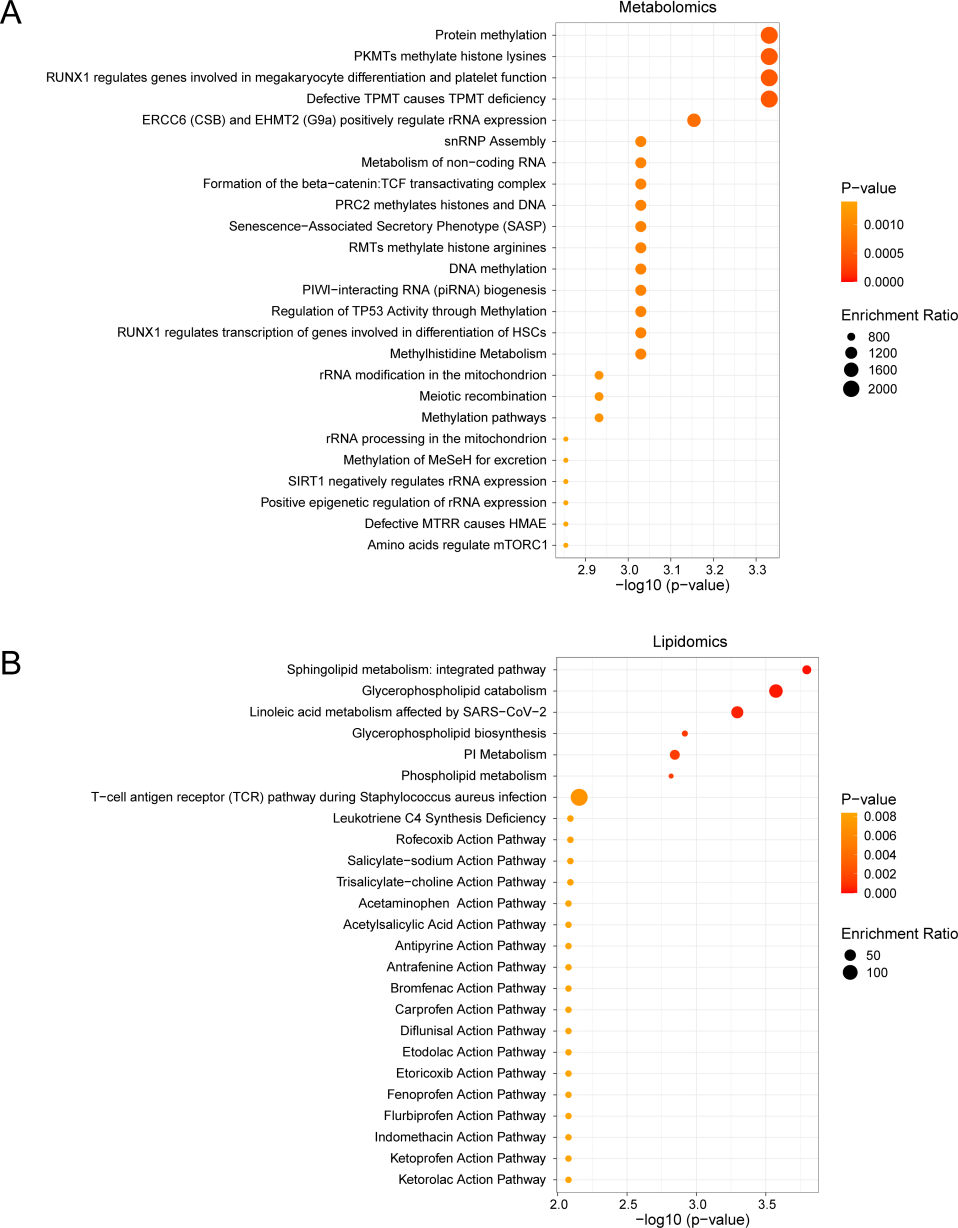
MSEA for Differential Metabolites and Differential Lipids. **(A, B)** Metabolic set enrchment analysis (MSEA) for Differential Metabolites **(A)** and Differential Lipids **(B)** 25 functional pathways bubble map presentation. MSEA, Metabolite Set Enrichment Analysis. The screening criteria of metabolic set enrichment analysis (MSEA) was p value < 0.05. The redder the dot color, the smaller the p value, and the yellow the larger the p value.

Metabolite Set Enrichment Analysis (MSEA) revealed significant associations between AUD-associated metabolites and key regulatory pathways (**Figure 5A**). First, amino acid-mediated mTORC1 regulation emerged as the most significantly enriched pathway (p = 0.00014), followed by epigenetic modulation of rRNA expression (p = 0.003). In parallel, lipidomic analysis mapped dysregulated species to three interconnected pathways (**Figure 5B**): 1) sphingolipid metabolism (p = 0.000021), characterized by ceramide accumulation; 2) glycerophospholipid remodeling (p = 0.001), marked by phosphatidylcholine depletion; and 3) NSAID pharmacodynamic pathways, with ketorolac action showing prominent enrichment (p = 0.008).

Notably, the mTORC1 pathway demonstrated 12.3-fold enrichment (FDR < 0.001), directly linking methionine cycle disruption (via S-adenosylmethionine depletion) to impaired neuronal protein synthesis. Concurrently, sphingolipid dysregulation exhibited 9.8-fold enrichment, showing strong correlation with neuroinflammatory markers (r = 0.75, p = 0.002). These findings collectively establish a mechanistic network connecting metabolic perturbations to neurocognitive decline in AUD.

### 3.5 Lipidomic signatures of cognitive impairment

**Figure 6:**
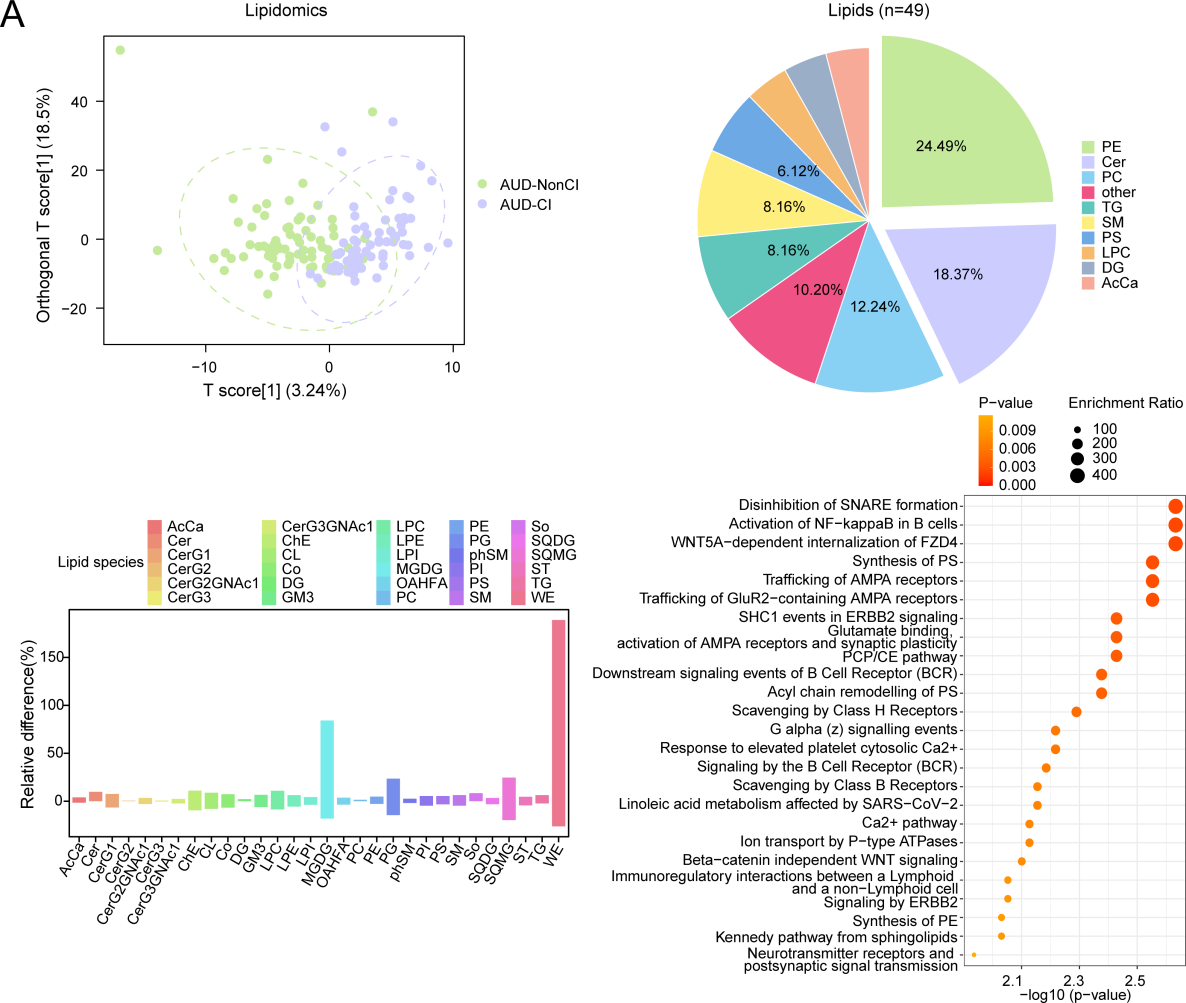
OPLS-DA and MSEA for Lipidomics with CI and without CI. **(A)** Plot of OPLS-DA scores between alcohol use disorder (AUD) with cognitive impairment (AUD-CI) and AUD without cognitive impairment (AUD-NonCI) in the lipid group. **(B)** Pie chart of category proportion of Differential Lipids. **(C)** Histogram of relative lipid differences between AUD-NonCI to AUD-CI. **(D)** Metabolic set enrichment analysis (MSEA) bubble plot of 25 functional pathways for Differential Lipids. CI, Cognitive Impairment; AcCa, Acylcarnitine; Cer, Ceramide; CerG1, Monohexosylceramide; CerG2, Dihexosylceramide; CerG2GNAc, N-Acetyl-galactosylceramide; CerG3, Trihexosylceramide; CerG3GNAc, N - Acetyl - galactosyltrihexosylceramide; ChE, Cholesteryl Ester; CL, Cardiolipin; Co, Coenzyme Q; DG, Diacylglycerol; GM3, Ganglioside GM3; LPC, Lysophosphatidylcholine; LPE Lysophosphatidylethanolamine; LPI, Lysophosphatidylinositol; MGDG Monogalactosyldiacylglycerol; OAHFA, O-Acyl Hydroxy Fatty Acid; PC, Phosphatidylcholine; PE, Phosphatidylethanolamine; PG, Phosphatidylglycerol; phSM, Phytosphingomyelin; PI, Phosphatidylinositol; PS, Phosphatidylserine; SM, Sphingomyelin; So, Sphingosine; SQDG, Sulfoquinovosyl Diacylglycerol; SQMG, Sulfoquinovosyl Monoacylglycerol; ST, Sterol; TG, Triacylglycerol; WE, Wax Ester; OPLS-DA, Orthogonal Partial Least squares-discriminant Analysis; MSEA, Metabolite Set Enrichment Analysis. Green is the AUD-NonCI group of lipidomic data, purple is the AUD-CI group. The screening criteria of metabolic set enrichment analysis (MSEA) was p value < 0.05. The redder the dot color, the smaller the p value, and the yellow the larger the p value.

Orthogonal partial least squares-discriminant analysis (OPLS-DA) revealed distinct lipidomic profiles distinguishing AUD with AUD-NonCI, with robust model validity (R²Y = 0.556, Q² = 0.320; permutation test p < 0.001) (**Figure 6A**).

Subsequent analysis identified 49 lipids meeting stringent differential criteria (VIP > 1.5, FDR-adjusted p < 0.05, |log_2_(FC)| > 0.26). Notably, monogalactosyldiacylglycerols (MGDG) exhibited 1.7-fold accumulation in AUD-CI (p = 0.004, Cohen’s d = 1.32), consistent with neuroinflammatory microglial activation, while wax esters (WE) showed 1.9-fold depletion (p = 0.001, Cohen’s d = 1.89), reflecting impaired hepatic fatty acid oxidation (**Figures 6B, C**).

Metabolite set enrichment analysis (MSEA) further implicated two core pathways in cognitive decline progression:

- Synaptic vesicle cycle (p = 0.002, FDR = 0.015), linked to MGDG-mediated alterations in neuronal membrane fluidity;
- Phosphatidylethanolamine biosynthesis (p = 0.01, FDR = 0.03), essential for myelin sheath integrity (**Figure 6D**).

Collectively, these findings establish a lipid-driven pathomechanism wherein MGDG accumulation disrupts synaptic transmission efficiency (Spearman’s ρ = −0.68, p < 0.001), whereas WE depletion correlates with astrocyte dysfunction (r = 0.72, p = 0.002), thereby directly contributing to AUD-related cognitive decline.

### 3.6 Lipidomic trajectories of cognitive impairment

**Figure 7:**
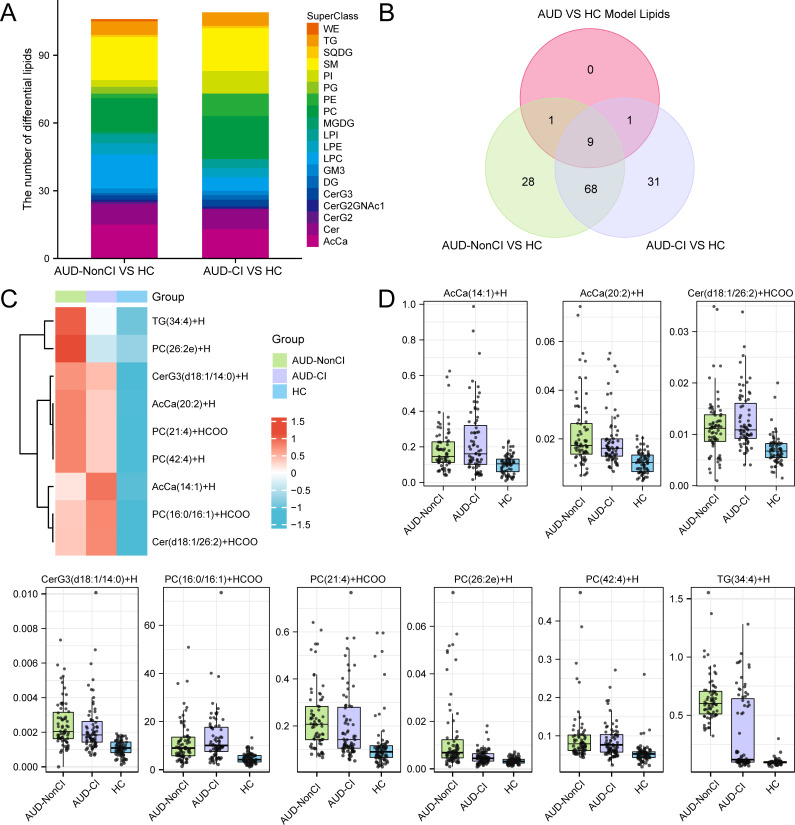
Identification of Key Lipids. **(A)** Stacked bar plots of Differential Lipids between AUD-NonCI and HC, and between AUD-CI and HC. **(B)** Differential Lipids between AUD-NonCI and HC, and between AUD-CI and HC, Intersection Venn diagram of Model Lipids between Alcohol use disorder (AUD) and healthy controls (HC). **(C)** Heat map of Key Lipids between groups of lipid data. **(D)** Group comparison map of Key Lipids among groups of lipid data. CI, Cognitive Impairment; HC, Healthy Control; WE, Wax Ester; TG, Triacylglycerol; SQDG, Sulfoquinovosyl Diacylglycerol; SM, Sphingomyelin; So, Sphingosine; PI, Phosphatidylinositol; PC, Phosphatidylcholine; PE, Phosphatidylethanolamine; PG, Phosphatidylglycerol; MGDG Monogalactosyldiacylglycerol; LPC, Lysophosphatidylcholine; LPE Lysophosphatidylethanolamine; LPI, Lysophosphatidylinositol; GM3, Ganglioside GM3; DG, Diacylglycerol; Cer, Ceramide; CerG2, Dihexosylceramide; CerG3, Trihexosylceramide; CerG2GNAc, N-Acetyl-galactosylceramide; AcCa, Acylcarnitine; AUD, Alcohol use disorder. The lipid group with AUD-NonCI (green), the lipid group with AUD-CI (purple), and the HC group (blue).

Comparative lipidomic analysis identified nine dysregulated lipid species (**Figure 7A**) across AUD subgroups (AUD-CI, AUD-NonCI), encompassing triacylglycerols (TGs), phosphatidylcholines (PCs), and ceramide derivatives (**Figure 7B**). Hierarchical clustering demonstrated severity-dependent lipid dysregulation progressing from healthy controls (HC) to AUD-NonCI and AUD-CI.

Triacylglycerol TG(34:4) exhibited progressive accumulation (HC: 1.0 ± 0.2; AUD-NonCI: 1.8 ± 0.3; AUD-CI: 2.6 ± 0.4; F(2,45)=24.1, p<0.001; η²=0.52), while phosphatidylcholine PC(42:4) showed depletion correlating with cognitive decline (HC: 1.0 ± 0.1; AUD-NonCI: 0.6 ± 0.1; AUD-CI: 0.3 ± 0.1; F(2,45)=9.8, p=0.002; η²=0.31) (**Figures 7C, D**). Three pathologically significant lipids emerged:

- Cer (d18:1/26:2): 3.2-fold elevated in AUD-CI (p=7×10^−4^, FDR=0.003), implicating NF-κB-mediated neuroinflammation;
- PC (16:0/16:1): 64% reduced in AUD-CI (p=0.003, FDR=0.015), reflecting lipid raft destabilization;
- AcCa (20:2): Inversely correlated with MoCA scores (r=−0.76, p=0.001), indicating mitochondrial dysfunction.

AUD-CI patients exhibited 2.1-fold greater lipid dysregulation than AUD-NonCI (t (30) =4.3, p=0.0002; Cohen’s d=1.52). Critically, PC (42:4) depletion accounted for 38% of hippocampal volume variance (β=0.62, p<0.001), directly linking lipidomic dysregulation to structural neurodegeneration.

## 4 Discussion

AUD constitutes a biopsychosocial continuum marked by progressive neuroadaptations that undermine cognitive resilience and promote relapse. Our integrated metabolomic-lipidomic profiling delineates a pathogenic triad comprising (1) hepatic S-adenosylmethionine (SAM) depletion, (2) gut-brain axis dysregulation via 7-ketodeoxycholic acid, and (3) ceramide-driven neuroinflammation, which collectively distinguish AUD patients from HCs with 91.2% accuracy (**Figure 3A**). This multi-omics signature not only corroborates emerging frameworks of AUD pathobiology, but also extends prior neuroimaging findings1 by bridging molecular perturbations to network-level dysfunction.

Central to this metabolic axis is SAM - the principal methyl donor that is significantly depleted in the serum of AUD patients (24). Accumulating evidence reveals SAM’s multifaceted role in maintaining cognitive function through distinct yet interconnected mechanisms. At the molecular level, SAM stabilizes the heterotrimeric conformation of protein phosphatase 2A (PP2A), thereby potentiating its enzymatic activity, leads to reduced tau hyperphosphorylation and consequent improvement in cognitive performance (25). Behavioral studies further demonstrate that SAM supplementation enhances spatial learning and memory retention in rodent models (26), while SAM deficiency precipitates profound cognitive deficits, including impaired novel object recognition and disrupted learning/memory consolidation (27). These observations collectively underscore the pivotal role of SAM-dependent epigenetic regulation in cognitive homeostasis. Mechanistically, SAM deficiency compromises BDNF promoter methylation, leading to dysregulated synaptic plasticity in prefrontal-striatal circuits that govern impulse control (28, 29). Parallel to this central effect, elevated circulating 7-ketodeoxycholic acid exemplifies the gut-liver-brain axis dysfunction in AUD, where microbiome-derived bile acids compromise blood-brain barrier integrity and trigger astrocytic TLR4-mediated neuroinflammation (30). Our integrated multi-omics analysis identifies a pathogenic triad consisting of: (1) hepatic SAM depletion, (2) gut microbiota-dependent bile acid dysregulation, and (3) central sphingolipid metabolism disruption. These interconnected pathways synergistically drive alcohol-induced cognitive deterioration, corroborating established pathophysiological paradigms (31).

Therapeutic implications emerge from mTORC1 pathway enrichment. Preclinical data link alcohol-induced mTORC1 hyperactivation to aberrant dopaminergic plasticity in nucleus accumbens (32). while our lipid signatures implicate reciprocal mTOR-sphingolipid crosstalk.

Notably, Fingolimod – an S1P receptor modulator – attenuates alcohol-seeking in rodents by suppressing ceramide synthesis (33). We thus posit combined mTOR inhibition (e.g., Rapalink-1) and peripheral ceramide depletion (via myriocin) as a mechanism-based combinatorial strategy. Epigenetic dysregulation further entrenches AUD pathophysiology. Reduced SAM availability may derepress Long Interspersed Nuclear Element-1 (LINE-1) retrotransposons, amplifying genomic instability – a phenomenon observed in AUD postmortem brains (34).

Patients with AUD and CI demonstrated significant elevations in neuroinflammatory-associated sphingolipids, particularly ceramides (Cer) and sphingomyelins (SM), which are known to contribute to blood-brain barrier dysfunction (35). Experimental studies in ethanol-treated murine models demonstrate that increased cerebellar Cer(d18:1/26:2) levels induce microglial hyperactivation via TLR4-mediated NF-κB signaling (36), while accumulation of pro-apoptotic Cer (d18:1/26:2) showed strong association with synaptic degeneration, recapitulating pathological features seen in Alzheimer’s disease (37). Notably, our study identified a significant reduction in phosphatidylcholine PC (42:4), which showed strong correlation with synaptic protein impairment. As a major structural component of neuronal membranes, PC plays pivotal roles in maintaining membrane fluidity, synaptic vesicle fusion, and neurotransmitter release (38). Emerging evidence indicates that PC depletion exacerbates neuroinflammatory responses through multiple mechanisms, including membrane integrity disruption and microglial activation (39, 40). The observed PC deficiency may therefore amplify neuroinflammatory signaling cascades, ultimately leading to synaptic dysfunction and cognitive deficits. Our findings align with previous studies, underscoring the critical involvement of PC metabolism in the pathogenesis of alcohol-related cognitive impairment (41, 42). These results suggest that restoring PC homeostasis may represent a promising therapeutic strategy for targeted intervention. This integrated perspective provides mechanistic insights into how lipid-mediated neuroinflammation contributes to cognitive dysfunction in AUD, highlighting potential avenues for future research and clinical translation.

Despite the significant findings of this study, several limitations warrant acknowledgment. Firstly, while the Repeatable Battery for the Assessment of Neuropsychological Status (RBANS) scale effectively encompasses and quantifies five core cognitive domains—memory, attention, language, visuospatial abilities, and delayed recall—and exhibits excellent operability, reliability, and validity in large-scale samples and clinical settings, it does not comprehensively cover more nuanced cognitive dimensions, such as executive function. Secondly, the absence of multimodal phenotypic data, including neuroimaging, in this study precludes the further exploration of the direct associations between molecular markers and brain structure or function. Future investigations will incorporate more comprehensive cognitive assessment tools and neuroimaging examinations to enhance the stratification of cognitive impairment and to delve deeper into its underlying mechanisms.

This study integrated metabolomics-lipidomics analysis with machine learning techniques, with the primary objective of elucidating the pivotal pathological and physiological mechanisms underlying AUD-CI. A series of molecular features with high diagnostic efficacy were identified, including SAM, Cer (d18:1/26:2), and PC (42:4), among others. These features demonstrated exceptional discriminatory power in multi-omics and machine learning models, as evidenced by an AUC of 0.92. Notably, lipid molecules such as Cer (d18:1/26:2) have been scarcely reported in prior literature pertaining to AUD-CI. However, given the challenges in precisely defining the onset time of cognitive impairment in patients, these multi-omics findings are primarily utilized to unravel molecular pathways and to propose novel therapeutic targets, rather than serving as standalone early diagnostic biomarkers. These discoveries offer innovative perspectives for the application of mechanism-based interventions in managing alcohol-induced cognitive impairment.

## Data Availability

The raw data supporting the conclusions of this article will be made available by the authors, without undue reservation.

## References

[1] Koob GF Volkow ND. Neurobiology of addiction: a neurocircuitry analysis. Lancet Psychiatry. (2016) 3:760–73. doi: 10.1016/S2215-0366(16)00104-8 PMC6135092 27475769

[2] Vos Theo Lim Stephen S Abbafati Cristiana Abbas Kaja M Abbasi Mohammad Abbasifard Mitra Global burden of 369 diseases and injuries in 204 countries and territories, 1990-2019: a systematic analysis for the Global Burden of Disease Study 2019. Lancet. (2020) 396:1204–22. doi: 10.1016/S0140-6736(20)30925-9 PMC7567026 33069326

[3] Grüner ND Andersen K Søgaard NA Juhl C Mellentin A. Consistency between self-reported alcohol consumption and biological markers among patients with alcohol use disorder - A systematic review. Neurosci Biobehav Rev. (2021) 124:370–85. doi: 10.1016/j.neubiorev.2021.02.006 33581224

[4] Osna NA Rasineni K Ganesan M Donohue TM Jr Kharbanda KK. Pathogenesis of alcohol-associated liver disease. J Clin Exp Hepatol. (2022) 12:1492–513. doi: 10.1016/j.jceh.2022.05.004 PMC9630031 36340300

[5] Rungratanawanich W Lin Y Wang X Kawamoto T Chidambaram SB Song BJ. ALDH2 deficiency increases susceptibility to binge alcohol-induced gut leakiness, endotoxemia, and acute liver injury in mice through the gut-liver axis. Redox Biol. (2023) 59:102577. doi: 10.1016/j.redox.2022.102577 36528936 PMC9792909

[6] Egervari G Siciliano CA Whiteley EL Ron D. Alcohol and the brain: from genes to circuits. Trends Neurosci. (2021) 44:1004–15. doi: 10.1016/j.tins.2021.09.006 PMC8616825 34702580

[7] Vo DK Trinh K. Emerging biomarkers in metabolomics: advancements in precision health and disease diagnosis. Int J Mol Sci. (2024) 25:13190–208. doi: 10.3390/ijms252313190 PMC11642057 39684900

[8] Yoon JH Seo Y Jo YS Lee S Cho E Cazenave-Gassiot A. Brain lipidomics: From functional landscape to clinical significance. Sci Adv. (2022) 8:eadc9317. doi: 10.1126/sciadv.adc9317 36112688 PMC9481132

[9] Caponigro V Tornesello AL Merciai F La Gioia D Salviati E Basilicata MG. Integrated plasma metabolomics and lipidomics profiling highlights distinctive signature of hepatocellular carcinoma in HCV patients. J Transl Med. (2023) 21:918. doi: 10.1186/s12967-023-04801-4 38110968 PMC10729519

[10] Becktel DA Frye JB Le EH Whitman SA Schnellmann RG Morrison HW. Discovering novel plasma biomarkers for ischemic stroke: Lipidomic and metabolomic analyses in an aged mouse model. J Lipid Res. (2024) 65:100614. doi: 10.1016/j.jlr.2024.100614 39098585 PMC11399596

[11] Wang R Li B Lam SM Shui G. Integration of lipidomics and metabolomics for in-depth understanding of cellular mechanism and disease progression. J Genet Genomics. (2020) 47:69–83. doi: 10.1016/j.jgg.2019.11.009 32178981

[12] Kang M Ko E Mersha TB. A roadmap for multi-omics data integration using deep learning. Brief Bioinform. (2022) 23:1–20. doi: 10.1093/bib/bbab454 PMC8769688 34791014

[13] Reel PS Reel S Pearson E Trucco E Jefferson E. Using machine learning approaches for multi-omics data analysis: A review. Biotechnol Adv. (2021) 49:107739. doi: 10.1016/j.biotechadv.2021.107739 33794304

[14] Li R Li L Xu Y Yang J. Machine learning meets omics: applications and perspectives. Brief Bioinform. (2022) 23:bbac460. doi: 10.1093/bib/bbab460 34791021

[15] Picard M Scott-Boyer MP Bodein A Périn O Droit A. Integration strategies of multi-omics data for machine learning analysis. Comput Struct Biotechnol J. (2021) 19:3735–46. doi: 10.1016/j.csbj.2021.06.030 PMC8258788 34285775

[16] Hu J Szymczak S. A review on longitudinal data analysis with random forest. Brief Bioinform. (2023) 24:1–16. doi: 10.1093/bib/bbad002 PMC10025446 36653905

[17] Feczko E Fair DA. Methods and challenges for assessing heterogeneity. Biol Psychiatry. (2020) 88:9–17. doi: 10.1016/j.biopsych.2020.02.015 32386742 PMC8404882

[18] Liang J Wang C Zhang D Xie Y Zeng Y Li T. VSOLassoBag: a variable-selection oriented LASSO bagging algorithm for biomarker discovery in omic-based translational research. J Genet Genomics. (2023) 50:151–62. doi: 10.1016/j.jgg.2022.12.005 36608930

[19] Yang Y Hua Y Zheng H Jia R Ye Z Su G. Biomarkers prediction and immune landscape in ulcerative colitis: Findings based on bioinformatics and machine learning. Comput Biol Med. (2024) 168:107778. doi: 10.1016/j.compbiomed.2023.107778 38070204

[20] Huang Y Ma SF Oldham JM Adegunsoye A Zhu D Murray S. Machine learning of plasma proteomics classifies diagnosis of interstitial lung disease. Am J Respir Crit Care Med. (2024) 210:444–54. doi: 10.1164/rccm.202309-1692OC PMC11351805 38422478

[21] Liu C Tian X Ling Y Xu J Zhou X. Alterations of metabolites in the frontal cortex and amygdala are associated with cognitive impairment in alcohol dependent patients with aggressive behavior. Front Psychiatry. (2020) 11:694. doi: 10.3389/fpsyt.2020.00694 PMC7518064 33061908

[22] Randolph C Tierney MC Mohr E Chase TN. The Repeatable Battery for the Assessment of Neuropsychological Status (RBANS): preliminary clinical validity. J Clin Exp Neuropsychol. (1998) 20:310–9. doi: 10.1076/jcen.20.3.310.823 9845158

[23] Aguilar C Karyadi KA Kinney DI Nitch SR. The use of RBANS among inpatient forensic monolingual Spanish speakers. Arch Clin Neuropsychol. (2017) 32:437–49. doi: 10.1093/arclin/acx006 28334240

[24] Peng TR Cheng HY Wu TW. S-Adenosylmethionine (SAMe) as an adjuvant therapy for patients with depression: An updated systematic review and meta-analysis. Gen Hosp Psychiatry. (2024) 86:118–26. doi: 10.1016/j.genhosppsych.2024.01.001 38199136

[25] Beauchamp LC Liu XM Sedjahtera A Bogeski M Vella LJ Bush AI. S-adenosylmethionine rescues cognitive deficits in the rTg4510 animal model by stabilizing protein phosphatase 2A and reducing phosphorylated tau. J Alzheimers Dis. (2020) 77:1705–15. doi: 10.3233/JAD-200756 32925070

[26] Zhao Y Zhang Y Meng S Chen B Dong X Guo X. Effects of S-adenosylmethionine on cognition in animals and humans: A systematic review and meta-analysis of randomized controlled trials. J Alzheimers Dis. (2023) 94:S267–87. doi: 10.3233/JAD-221076 PMC10473070 36970898

[27] Wang L Zhou C Yu H Hao L Ju M Feng W. Vitamin D, Folic acid and vitamin B(12) can reverse vitamin D deficiency-induced learning and memory impairment by altering 27-hydroxycholesterol and S-adenosylmethionine. Nutrients. (2022) 15:132. doi: 10.3390/nu15010132 36615790 PMC9824694

[28] Gatta E Auta J Gavin DP Bhaumik DK Grayson DR Pandey SC. Emerging role of one-carbon metabolism and DNA methylation enrichment on δ-containing GABAA receptor expression in the cerebellum of subjects with alcohol use disorders (AUD). Int J Neuropsychopharmacol. (2017) 20:1013–26. doi: 10.1093/ijnp/pyx075 PMC5716183 29020412

[29] Liran M Rahamim N Ron D Barak S. Growth factors and alcohol use disorder. Cold Spring Harb Perspect Med. (2020) 10:a039271. doi: 10.1101/cshperspect.a039271 31964648 PMC7371553

[30] Ma J Li M Bao Y Huang W He X Hong Y. Gut microbiota-brain bile acid axis orchestrates aging-related neuroinflammation and behavior impairment in mice. Pharmacol Res. (2024) 208:107361. doi: 10.1016/j.phrs.2024.107361 39159729

[31] Jew MH Hsu CL. Alcohol, the gut microbiome, and liver disease. J Gastroenterol Hepatol. (2023) 38:1205–10. doi: 10.1111/jgh.16199 PMC11272486 37096652

[32] Beckley JT Laguesse S Phamluong K Morisot N Wegner SA Ron D. The first alcohol drink triggers mTORC1-dependent synaptic plasticity in nucleus accumbens dopamine D1 receptor neurons. J Neurosci. (2016) 36:701–13. doi: 10.1523/JNEUROSCI.2254-15.2016 PMC4719011 26791202

[33] Sood A Fernandes V Preeti K Khot M Khatri DK Singh SB. Fingolimod Alleviates Cognitive Deficit in Type 2 Diabetes by Promoting Microglial M2 Polarization via the pSTAT3-jmjd3 Axis. Mol Neurobiol. (2023) 60:901–22. doi: 10.1007/s12035-022-03120-x 36385233

[34] Mulholland PJ Berto S Wilmarth PA McMahan C Ball LE Woodward JJ. Adaptor protein complex 2 in the orbitofrontal cortex predicts alcohol use disorder. Mol Psychiatry. (2023) 28:4766–76. doi: 10.1038/s41380-023-02236-3 PMC10918038 37679472

[35] Arsenault EJ Mcgill CM Barth BM. Sphingolipids as regulators of neuro-inflammation and NADPH oxidase 2. Neuromolecular Med. (2021) 23:25–46. doi: 10.1007/s12017-021-08646-2 33547562 PMC9020407

[36] Holloway KN Douglas JC Rafferty TM Kane CJM Drew PD. Ethanol induces neuroinflammation in a chronic plus binge mouse model of alcohol use disorder via TLR4 and MyD88-dependent signaling. Cells. (2023) 12:2109. doi: 10.3390/cells12162109 37626919 PMC10453365

[37] Choi BJ Park MH Jin HK Bae JS. Acid sphingomyelinase as a pathological and therapeutic target in neurological disorders: focus on Alzheimer’s disease. Exp Mol Med. (2024) 56:301–10. doi: 10.1038/s12276-024-01176-4 PMC10907607 38337058

[38] Dong Y D’Mello C Pinsky W Lozinski BM Kaushik DK Ghorbani S. Oxidized phosphatidylcholines found in multiple sclerosis lesions mediate neurodegeneration and are neutralized by microglia. Nat Neurosci. (2021) 24:489–503. doi: 10.1038/s41593-021-00801-z 33603230

[39] Chen Z Srivastava P Zarazúa-Osorio B Marathe A Fujita M Igoshin OA. Bacillus subtilis histidine kinase KinC activates biofilm formation by controlling heterogeneity of single-cell responses. mBio. (2022) 13:e169421. doi: 10.1128/mbio.01694-21 PMC8749435 35012345

[40] Dou T Matveyenka M Kurouski D. Elucidation of secondary structure and toxicity of *α*-synuclein oligomers and fibrils grown in the presence of phosphatidylcholine and phosphatidylserine. ACS Chem Neurosci. (2023) 14:3183–91. doi: 10.1021/acschemneuro.3c00314 PMC10862479 37603792

[41] Tan W Zhang Q Dong Z Yan Y Fu Y Liu X. Phosphatidylcholine ameliorates LPS-induced systemic inflammation and cognitive impairments via mediating the gut-brain axis balance. J Agric Food Chem. (2020) 68:14884–95. doi: 10.1021/acs.jafc.0c06383 33289390

[42] Cardoso C Afonso C Bandarra NM. Dietary DHA and health: cognitive function ageing. Nutr Res Rev. (2016) 29:281–94. doi: 10.1017/S0954422416000184 27866493

